# Extremely Long-Lived Stigmas Allow Extended Cross-Pollination Opportunities in a High Andean Plant

**DOI:** 10.1371/journal.pone.0019497

**Published:** 2011-05-05

**Authors:** Cristian Torres-Díaz, Susana Gómez-González, Gisela C. Stotz, Patricio Torres-Morales, Brayam Paredes, Matías Pérez-Millaqueo, Ernesto Gianoli

**Affiliations:** 1 Laboratorio de Genómica and Biodiversidad (LGB), Departamento de Ciencias Básicas, Universidad del Bío-Bío, Chillán, Chile; 2 Departamento de Botánica, Universidad de Concepción, Concepción, Chile; 3 Center for Advanced Studies in Ecology and Biodiversity (CASEB), P. Universidad Católica de Chile, Santiago, Chile; 4 Departamento de Biología, Universidad de La Serena, La Serena, Chile; Trinity College Dublin, Ireland

## Abstract

High-elevation ecosystems are traditionally viewed as environments in which predominantly autogamous breeding systems should be selected because of the limited pollinator availability. *Chaetanthera renifolia* (Asteraceae) is an endemic monocarpic triennial herb restricted to a narrow altitudinal range within the high Andes of central Chile (3300–3500 m a.s.l.), just below the vegetation limit. This species displays one of the larger capitulum within the genus. Under the reproductive assurance hypothesis, and considering its short longevity (monocarpic triennial), an autogamous breeding system and low levels of pollen limitation would be predicted for *C. renifolia*. In contrast, considering its large floral size, a xenogamous breeding system, and significant levels of pollen limitation could be expected. In addition, the increased pollination probability hypothesis predicts prolonged stigma longevity for high alpine plants. We tested these alternative predictions by performing experimental crossings in the field to establish the breeding system and to measure the magnitude of pollen limitation in two populations of *C. renifolia*. In addition, we measured the stigma longevity in unpollinated and open pollinated capitula, and pollinator visitation rates in the field. We found low levels of self-compatibility and significant levels of pollen limitation in *C. renifolia*. Pollinator visitation rates were moderate (0.047–0.079 visits per capitulum per 30 min). Although pollinator visitation rate significantly differed between populations, they were not translated into differences in achene output. Finally, *C. renifolia* stigma longevity of unpollinated plants was extremely long and significantly higher than that of open pollinated plants (26.3±2.8 days vs. 10.1±2.2, respectively), which gives support to the increased pollination probability hypothesis for high-elevation flowering plants. Our results add to a growing number of studies that show that xenogamous breeding systems and mechanisms to increase pollination opportunities can be selected in high-elevation ecosystems.

## Introduction

High-elevation environments are characterized by low temperatures, strong winds, and overcast conditions, which make them unsuitable for insect pollinators [1]. In these ecosystems, several community studies have documented that the levels of diversity, availability and activity of insect pollinators suffer progressive reductions with elevation above the timberline as a consequence of harsh climatic conditions that limit insect flight [2]–[9].

The reproductive assurance hypothesis posits that, for successful sexual reproduction to occur, autogamous reproduction and self-fertilization should evolve where pollinators are scarce [10]–[12]. Thus, transitions toward autogamous self-fertilization have been proposed as an evolutionary solution for alpine and arctic plants that deal with low pollinator availability. While some studies have found increases in self-fertilization with elevation [13], [14], there is also evidence of increased outcrossing [15] and decreased selfing rates with elevation [16]. In addition, the scarce pollinator service at high elevation has been argued as a cause to explain the high frequency of asexual reproduction (clonality and apomixis) in alpine species [17], [18]. Self-fertilization is also associated with adult longevity. For instance, self-fertilization is far more common in annuals than in perennials [19], [20].

In contrast to the reproductive assurance hypothesis, the increased pollination probability hypothesis predicts that increases in flower showiness [17], [21], [22] and flower longevity with elevation [23]–[29] would compensate for the scarcity of pollinators at higher altitudes, challenging the traditional assumption that biotic pollination is limited in high-elevation environments. In general, pollen limitation (the reduction in reproductive success because of a shortage in pollen supply) in obligate out-crossing species tends to be higher than in self-compatible species [30]. Due to the low pollinator abundance, pollen limitation is expected to be high in high-elevation environments [e.g. 31, 32]. A recent meta-analysis by García-Camacho & Totland (2009) [33] reported that although alpine plants show significant pollen limitation, there is no difference in pollen limitation between alpine and lowland species.

*Chaetanthera renifolia* (Asteraceae) is an endemic species that has been historically restricted to a narrow elevation range (3200–3400 m asl) within the high Andes of the province of Santiago, in central Chile [34]. *Chaetanthera renifolia* has one of the largest capitula among high-elevation Andean species within the genus (Table 1) [35] and is also characterized by its short adult longevity, being a monocarpic triennial (Torres-Díaz, unpublished data). The aims of the present study were to determine the breeding system, the magnitude of pollen limitation, pollinator visitation rates, and the stigma longevity in *C. renifolia*. Considering the reproductive assurance hypothesis and its short adult longevity, a predominantly autogamous breeding system with low levels of pollen limitation would be expected for *C. renifolia*. In contrast, under the increased pollination probability hypothesis, the large *C. renifolia* capitulum size within its genus (Table 1) could be interpreted as an adaptation to deal with low pollinator availability and hence an outcrossing breeding system, with the high pollen limitation and high stigma longevity that could be expected for this species.

**Table 1:** Capitulum and disc size (mean ± SD) of *Chaetanthera renifolia* and other coexisting congeners at the same Andean basin in central Chile.

Species	Capitulum size (mm)	Disc size (mm)
*Chaetanthera renifolia*		
La Parva (NW, 3340) *n* = 50	46.3±5.14	16.8±4.03
Piedra Numerada (NE, 3460) *n* = 50	44.0±5.12	15.3±2.45
*Chaetanthera pentacaenoides*		
La Parva (W, 3300) *n* = 50	8.5±0.70 *	2.5±0.32 *
Tres puntas (Flat, 3600) *n* = 50	7.9±0.63 *	2.1±0.31 *
*Chaetanthera apiculata*		
La Parva (N, 3160) *n* = 50	18.9±1.76 *	5.1±0.57 *
La Parva (W, 3100) *n* = 50	20.3±2.30 *	5.7±0.78 *
*Chaetanthera lycopodioides*		
La Parva (W, 3250) *n* = 50	14.3±1.78 *	3.2±0.57 *
La Terraza (E, 3300) *n* = 50	15.3±1.47 *	3.2±0.45 *

The slope aspect, elevation (m asl) and number of sampled capitula are indicated for each population.

*Indicates significant differences (*P*<0.001, *t*-test) for comparisons of *C. renifolia* floral traits vs. each of the species.

## Methods

### The species

*Chaetanthera renifolia* (J. Rémy) Cabrera (Asteraceae: Mutisieae) is a small (3–4 cm tall) perennial (triennial) prostrate rosette herb, endemic to the high Andes of the Santiago province, Chile [34]. This species is branchless and is characterized by dark green renifom leaves. *C. renifolia* has sessile floral capitula (ca. 36 mm diameter) formed by white sterile ray flowers and bisexual yellow tubular disk flowers. *C. renifolia* normally displays only one capitulum per plant; individuals with more than one capitulum can also be found, yet in very low frequencies (<0.1%, C. Torres-Díaz, unpublished data). As many other Asteraceae species, *C. renifolia* is a protandrous herb (*i.e.,* anthers release pollen before stigmas are receptive) (C. Torres- Díaz, personal observation). This species shows broad variation in total capitulum size and disk diameter (Table 1). To date, individuals from only five populations of C. *renifolia* have been collected and stored in herbaria [36]. Due of the inherent difficulty of vegetation samplings in the high Andes (lack of roads), the conservation status of this species is yet to be determined. *C. renifolia* is at present regarded as a rare/insufficiently known species.

### Study sites

The present field study was conducted in two of the five currently known populations of *C. renifolia*, La Parva and Piedra Numerada, which are located within the subnival vegetation belt *sensu* Arroyo *et al.* (1981) [37], over 1100 m above the *Kageneckia angustifolia* (Rosaceae) tree-line at 2200 m asl. Mean annual temperature in the study area is 1.8–2.4°C [38]. The climate is alpine with influence from the Mediterranean-type climate existing in lowlands [39]. Mean annual precipitation, which mostly falls as snow during winter months, is 400–900 mm [40]. La Parva population (LP; 3360 m asl) is located on a NW slope (33°19′ 06.2″ S; 70°16′ 50.2″ W). Piedra Numerada population (PN; 3450 m asl) is located on a NE slope (33°17′49.2″ S; 70°13′18.5″ W). Linear geographic distance between the populations is *ca*. 6 km. Plant density did not differ between sites (mean number of plants per m^2^ ± 1 SE  = 0.638 ± 0.061 at LP and 0.599 ± 0.077 at PN; One-way ANOVA, *F*_1,100_ = 0.158, *P* = 0.691). The vegetation corresponds to the subnival Andean vegetation belt, which is dominated by sub-shrubs of the Asteraceae family (e.g., *Nassauvia* and *Senecio* spp.), cushion plants (e.g., *Laretia acaulis*), grasses (e.g., *Stipa* and *Hordeum comosum*) and rosette-forming small perennial herbs (e.g., *Viola* spp., *Pozoa* spp., *Adesmia* spp. and *Tropaeolum*) [38].

### Breeding system

To evaluate the breeding system of *C. renifolia*, a series of field experiments was carried out between January and April 2009 at LP and PN populations. We applied two pollination treatments (spontaneous self-pollination vs. hand cross-pollination) to evaluate the degree of dependence of *C. renifolia* on pollinators for successful achene production. Because of the small size (6–9 mm long) and the high number of florets per capitulum (50–250), it was not possible to emasculate florets to assess apomictic achene production. Achene production through geitonogamous self-fertilization among florets from different capitula of the same plant seems to be uncommon because of the very low abundance (<0.1%) of plants with more than one capitulum per plant (C. Torres-Díaz, field observation). Florets in Asteraceae usually bloom sequentially within capitula from outermost to innermost. Since each floret is protandous some overlapping between florets in male and female phases within capitulum can occurs. Thus, some geitonogamous self-pollination is possible when female-phase florets (from outermost) overlap with male-phase florets (from innermost). The spontaneous self-pollination treatment consisted of 18 plants excluded from insect pollinators by fine mesh nylon bags (1 mm mesh) just before anthesis. The hand cross-pollination treatment consisted of 18 focal plants that were manually crossed with fresh pollen from 5–7 individuals in male phase. Pollen was carefully brushed on the receptive stigmas of each of the 18 female-phase capitula. To avoid the possibility of reductions in fitness due to bi-parental inbreeding, pollen donors were located at least 10 meters apart from focal females. After withering, all sampled capitula (all plants displayed one capitulum) were covered with cloth bags to prevent losses of achenes and florets (from late February to March 2009). All bags were retrieved in March-April, allowing enough time for achene development. Each plant was analyzed for achene output (number of achenes) and achene quality (weight of achenes). We expressed achene output as percentage of achene set, which was measured as the percentage of ovaries of open florets that set achenes (100× (number of achenes/number of florets)). Given the difficulty of emasculating florets in Asteraceae, some degree of self-pollination could occur in hand pollination treatment. Therefore achene output of cross-pollination treatment was corrected subtracting the mean value of achene output of the spontaneous self-pollination treatment. In addition, we individually weighed ten dry achenes per capitulum to the nearest milligram using a digital balance. Finally, we calculated an autofertility index (AI) by dividing the achene set of bagged capitula by the achene set of hand-outcrossed capitula [12].

### Pollen limitation

To evaluate whether the amount of pollen reaching the stigmas constrains achene production of *C. renifolia*, we performed supplemental hand-pollination experiments. Along the flowering peak (between late January and early February), a total of 60 plants were sampled at each of the two populations (LP and PN). Flowering plants were randomly assigned either to supplemental hand cross-pollination (*n* = 30) or to control (*n* = 30) treatments. The controls consisted of untreated open-pollinated plants. Experimental plants were located at least 4 m apart from each other. Unfortunately, domestic livestock destroyed two plants from the supplemental hand-pollination treatment in PN population. All experiments were done in *C. renifolia* plants displaying one capitulum only. Supplemental hand-pollination was performed twice (in two different days) once floral capitula were at the female stage. Pollen addition was done by collecting pollen grains from 8–10 plants in male phase and carefully brushing them on receptive stigmas. Because crossing with relatives may reduce female reproductive success, pollen was collected from plants separated by at least 5 m. After withering, all capitula were carefully bagged with cloth sacs to retain achenes and florets. As in the breeding system experiments, we expressed achene output as the percentage of achene set. In addition, the mean achene weight (g) of individual achenes was calculated for each plant. To estimate mean achene weight a total of ten dry achenes were weighed to the nearest milligram using a digital balance.

### Pollinator visitation rates, flower visitors and female reproductive success

We estimated pollinator activity in *C. renifolia* at the peak of the flowering season (late January) [41]. Three independent observers simultaneously monitored insect visitation at three randomly chosen points within populations during periods of 30 min over a total of three sunny days per site. Data from all observers were pooled to obtain a single estimate of capitula visitation rate per each 30 min period, and six observation periods were obtained each day. Thus, the total observation time per plant per site was 540 min. Observations were made on all plants inside 3×3 m patches from 11:30 to 16:00. For each 30 min period the total number of open capitula within patches and air temperature (20 cm above ground level) were recorded. A flower visitor was counted as a pollinator only if it touched any disk floret. Pollinators were identified in the field and some were captured to ensure their identification at the species level and to verify the presence of pollen grains in their bodies. In order to evaluate whether potential differences in pollinator visitation rates between populations translate into differences in female reproductive success, achene output was estimated as described above.

### Stigma longevity

We quantified the stigma longevity of: (1) open-pollinated capitula and (2) bagged capitula (pollinator exclusion). A total of 36 capitula per population were randomly assigned to the two treatments (*n* = 18). Previous field observations (C. Torres-Díaz) indicate that *C. renifolia* is a protandrous species, with styles elongating upwards 1–2 days after the end of pollen dehiscence. The four-lobed shape of the stigma indicates the onset of stigma receptivity. Because successful fertilization of female florets rapidly induces style retraction, we consider that a floret remains receptive if the style persists elongated (upward) and stigma lobes remain opened. This operational criterion was used to estimate the duration of stigma receptivity (number of days). The measure of stigma longevity considers the longevity of all stigmas in a single capitulum. In order to evaluate whether the stigmas of capitula excluded from pollinators remain functional afterwards, i.e., maintaining their potential to set achenes, we performed hand-crossings in January of 2010. A total of 14 plants were bagged at the end of the male phase (80–90% of the florets) and were hand cross-pollinated 12 days after stigmas emerged from florets. After hand-pollination, experimental capitula were bagged again until plants withered. Finally, floral capitula were bagged as in breeding system experiments to retain florets and achenes.

### Statistical analyses

To test whether pollination treatment (spontaneous self-pollination vs. hand cross-pollination) and population (LP and PN) affected achene production and achene quality of *C. renifolia* we performed factorial ANCOVA's. Plant diameter (mm) was entered as a covariate to remove potential effects of differences in plant size between pollination treatments and sites. Differences in achene output and quality in control and supplemental pollination plants among populations were analyzed using factorial ANCOVA's and plant size (mm) as covariate. The percentage of achene set data were arcsine transformed to achieve a normal distribution both in breeding system and pollen limitation experiments. Comparison of pollinator visitation rates between populations was done using ANCOVA. Mean air temperature (20 cm above ground level) over each observation period was entered as a covariate to account for potential differences in microclimatic conditions between sites, which could promote differences in pollinator visitation rates, and might also influence how long it takes for achenes develop and flowering phenology. Differences in female reproductive success of plants from different populations were analyzed by ANCOVA, using the percentage of achene set as response variable, population as fixed factor and plant size as covariate. The effects of pollination (open-pollinated vs. pollinator excluded) and population (LP vs. PN) on stigma longevity were analyzed using a two-way ANOVA. In all cases, *post hoc* comparisons were made with Tukey tests. All analyses were performed using Statistica 6.0.

## Results

### Breeding system

**Figure 1:**
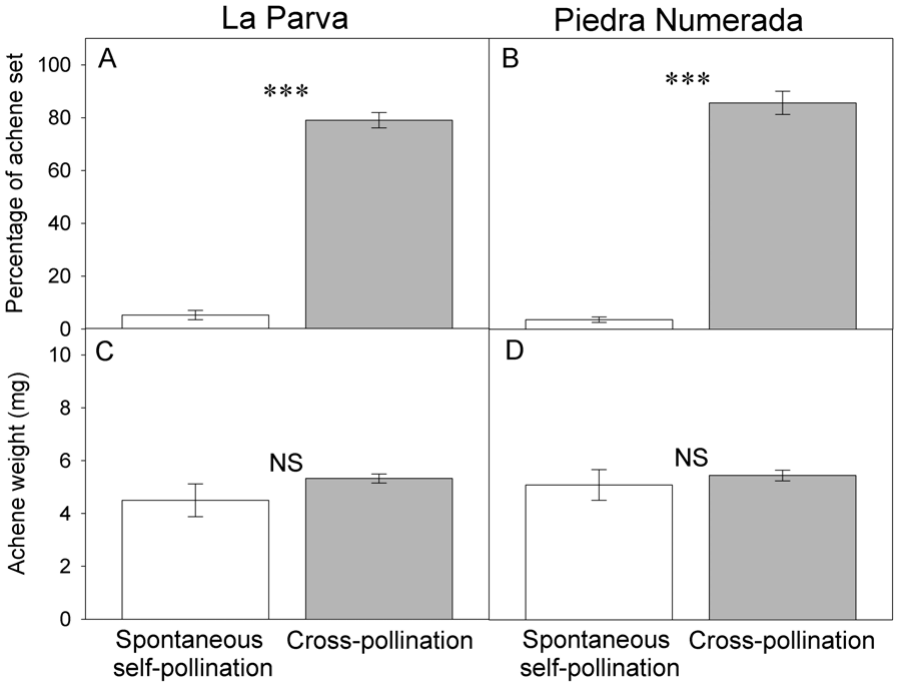
Effects of pollination treatments (spontaneous self-pollination vs. hand cross-pollination) on reproduction of *Chaetanthera renifolia*. Mean percentage of achene set and achene weight at La Parva (A and C) and Piedra Numerada (C and D). Open bars: autogamous self-pollinated (*n* = 18); Grey bars: hand cross-pollination (*n* = 18). Bars are means ± SE. *** Indicates significant differences (*P*<0.001).

Pollination treatment significantly affected the percentage of achene set of *C. renifolia* (Table 2). Both populations showed a low potential for autonomous self-fertilization (Autofertility-Index  = 0.041 and 0.067 for LP and PN, respectively). The percentage of achene set was 23.8 and 14.8 times higher in hand cross-pollinated than in spontaneously self-pollinated plants at LP and PN, respectively (Fig. 1A, B; Table 2). In contrast, achene weight did not differ between hand-cross pollination and spontaneous self-pollination treatments (Fig. 1C, D; Table 3). We did not find any significant interaction between pollination treatment and population (Table 2).

**Table 2:** Effects of pollination treatment on the reproduction of two populations of *Chaetanthera renifolia*.

Source of variation	DF	MS	F	P-value
*Percentage of achene set*				
Plant size (covariate)	1	0.01	0.58	0.499
Treatment (T)	1	12.47	385.41	<0.001
Site (S)	1	0.05	1.46	0.230
T x S	1	0.01	0.09	0.754
Error	67	0.04		
*Achene weight*				
Plant size (covariate)	1	1.43	0.39	0.529
Treatment (T)	1	2.61	0.73	0.396
Site (S)	1	3.47	0.97	0.328
T x S	1	1.59	0.45	0.507
Error	67	3.57		

ANCOVA summary of effects of pollination treatment (autonomous self-pollination vs. hand cross-pollination) and site (La Parva and Piedra Numerada) on the percentage of achene set and achene weight of *Chaetanthera renifolia* plants. Plant size (diameter) was entered as a covariate. Significant effects are indicated in bold.

**Table 3:** Effect of supplemental hand-pollination on the reproduction of two populations of *Chaetanthera renifolia.*

Source of variation	DF	MS	F	P-value
*Percentage of achene set*				
Plant size (covariate)	1	0.31	4.47	0.036
Treatment (T)	1	5.18	74.58	<0.001
Site (S)	1	0.11	1.57	0.212
T x S	1	0.01	0.08	0.771
Error	113	0.07		
*Achene weight*				
Plant size (covariate)	1	14.21	18.12	<0.001
Treatment (T)	1	75.89	96,77	<0.001
Site (S)	1	0.44	0.56	0.455
T x S	1	2.23	2.85	0.094
Error	113	0.78		

ANCOVA summary of the effects of pollination treatment (open-pollinated vs. supplemental hand pollination) and site (La Parva and Piedra Numerada) on the percentage of achene set and achene weight of *Chaetanthera renifolia* plants. Plant size (diameter) was entered as a covariate. Significant effects are indicated in bold.

### Pollen limitation

**Figure 2:**
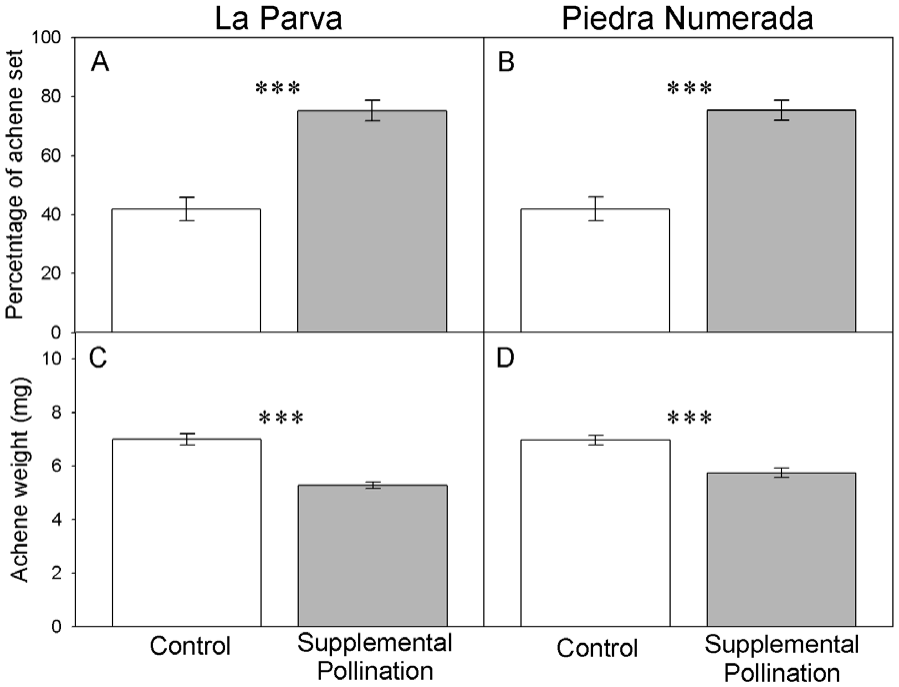
Effects of pollination treatments (control vs. supplemental hand-pollination) on reproduction of *Chaetanthera renifolia*. Mean percentage of achene set (A and B) and achene weight (mg) (C and D) at La Parva and Piedra Numerada. Open bars: control (*n* = 30); Grey bars: hand supplementary pollinated (*n* = 30). Bars are means ± SE. *** Indicates significant differences (*P*<0.001).

Supplemental hand-pollination resulted in a significant increase in achene production at both sites (Fig. 2, Table 3). The percentage of achene set was 33.4% and 34.3% higher in supplemental hand-pollinated than in control plants from LP and PN populations (*P*<0.001 in both cases, Tukey tests; Fig. 2A, B; Table 3). In contrast, supplemental hand cross-pollination resulted in a significant reduction in achene weight compared with open-pollinated capitula (1.7 and 1.2 mg in LP and PN, *P*<0.001 in both cases, Tukey tests; Fig. 2C, D; Table 3). We did not find any significant interaction between supplemental pollination and site for percentage of achene set nor for achene weight. The percentage of achene set and achene weight of open-pollinated plants from both sites did not differ (*P* = 0.835 and 0.998, respectively, Tukey tests).

### Pollinator assemblage, visitation rates and female reproductive success

**Figure 3:**
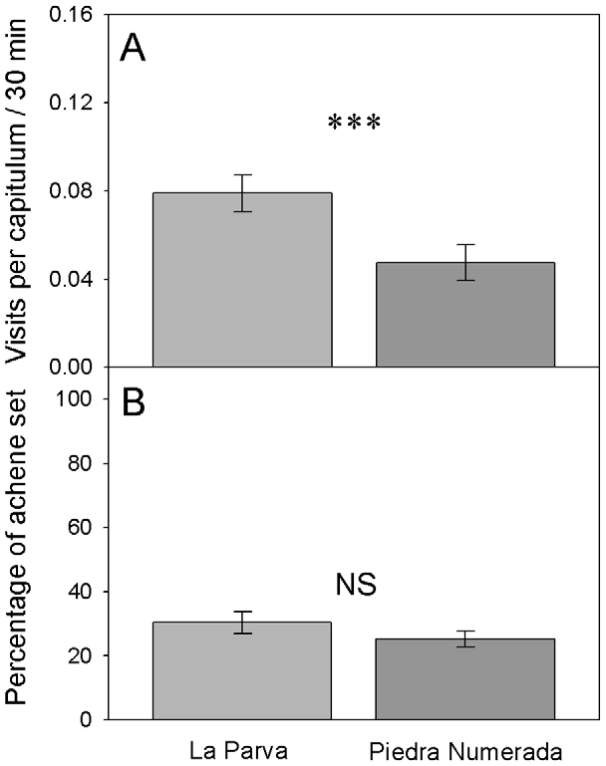
Pollinator visitation rates and achene output of two populations of *Chaetanthera renifolia*. (A) Mean pollinator visitation rate at La Parva (light-grey bars, *n* = 18) and Piedra Numerada (dark-grey bars, *n* = 17). (B) Mean percentage of achene set at La Parva (n = 40) and Piedra Numerada (*n* = 41). Bars are means ± SE. *** Indicates significant differences (*P*<0.01).

A total of 12 and 3 species of insects were observed on *C. renifolia* capitula at LP and PN, respectively (Table 4). While plants from LP were mainly visited by coleopterans and dipterans, individuals from PN were mainly pollinated by lepidopterans (Table 4). Air temperature did not differ between LP and PN (mean ±1 SE, 22.3±1.38°C at LP, 24.7±0.31°C at PN; *F*_1,33_ = 2.66, *P* = 0.112, One-way ANOVA). Pollinator visitation rate was significantly higher in LP than in PN (Fig. 3A, Table 5), thus suggesting a greater insect availability in the former population. However, the difference in pollinator visitation rates between sites did not result in differences in the percentage of achene set (Fig. 3B, Table 5).

**Table 4:** List and frequencies of insect pollinator taxa observed on *Chaetanthera renifolia* capitula.

Order/Family	Species	La Parva		Piedra Numerada	
		*N*	%	*N*	%
**Lepidoptera**					
* *Nymphalidae	*Faunula leucoglene*			34	72.3
	*Yramea lathonioides*	2	3.4		
**Diptera**					
Bombyliidae	*Villa* sp.	2	3.4	2	4.3
	*Villa gayi*	2	3.4		
	*Villa semifuscata*	2	3.4		
	*Sericosoma irwini*	4	6.8		
	*Lyophlaeba* sp.	2	3.4		
	*Triploechus bellus*	1	1.7		
Tachinidae	Tachinidae sp.	11	18.6	11	23.4
** **Syrphidae	*Scaeva melanostoma*	2	3.4		
**Hymenoptera**					
Megachilidae	*Megachile* sp.	2	3.4		
Andrenidae	*Lipanthus* sp.	5	8.5		
**Coleoptera**					
Curculionidae	*Arthrobrachus* sp.	24	40.7		
Total		59	100%	47	100%
Total number of taxa		12		3	

Number of individuals of each taxon observed (*N*), and percentage (%) of the total number of visits that each pollinator taxon made to *Chaetanthera renifolia* capitula from La Parva and Piedra Numerada.

**Table 5:** Effects of site on pollinator visitation rates and achene output of *Chaetanthera renifolia* capitula.

Source of variation	DF	MS	F	P-value
*Pollinator visitation rate*				
Temperature (covariate)	1	0.00	0.66	0.423
Site	1	0.01	7.83	**0.008**
Error	32	0.01		
*Percentage of achene set*				
Plant size (covariate)	1	774.29	2.07	0.154
Site	1	218.05	0.58	0.448
Error	77	374.92		

ANCOVA summary of the effects of site (La Parva and Piedra Numerada) on pollinator visitation rate (visits per flower capitulum/30 min) to *Chaetanthera renifolia* capitula and achene output. Air temperature (20 cm above soil surface) was entered as a covariate. Significant effects are indicated in bold.

### Stigma longevity

**Figure 4:**
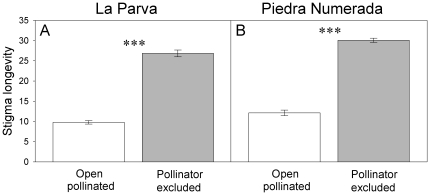
Effect of pollination (unpollinated vs. open-pollinated) on stigma longevity of *Chaetanthera renifolia*. Mean stigma longevity of open-pollinated (white bars, *n* = 18) and pollinator–excluded plants (grey bars, *n* = 18) at La Parva (A) and Piedra Numerada (B) populations of *Chaetanthera renifolia*. Bars are means ± SE. *** Indicates significant differences (*P*<0.01).

The stigmas of *C. renifolia* plants excluded from pollinators remained receptive for a significantly longer time (mean ± SE, LP = 25.8±1.9 days and PN = 26.8±3.5 days) than those of open-pollinated plants (LP = 10.4±2.8 days, PN = 9.8±2.2 days) (Fig. 4A, B; Table 6). There was no interaction between treatment and site (Table 6). The stigmas of plants excluded from pollinators remained functional at least 12 days after the onset of exclusion, as evidenced by a high achene production (mean ±1 SE; 56.0% ±3.93; *n* = 14) once these bagged capitula were hand-pollinated.

**Table 6:** Effects of pollination treatment and site on stigmas longevity of *Chaetanthera renifolia*.

Source of variation	DF	MS	F	P-value
Treatment (T)	1	4736.89	727.11	**<0.001**
Site (S)	1	0.89	0.14	0.721
T x S	1	12.5	1.91	0.170
Error	68	6.51		

Two-way ANOVA of the effects of treatment (pollinator-excluded vs. open-pollinated) and site (La Parva and Piedra Numerada) on stigma longevity (# days) of *Chaetanthera renifolia* capitula. Significant effects are indicated in bold.

## Discussion

Our results indicate that the endemic high Andean *C. renifolia* is a xenogamous, insect-pollinated species with low potential for autogamous seed production, and whose reproductive success is limited by pollen. Moreover, as predicted by the increased pollination probability hypothesis, *C. renifolia* showed high stigma longevity. As *C. renifolia* mostly reproduce through outcrossing, the extended stigma longevity would allow higher xenogamous pollination opportunities to this showy monocarpic high Andean plant.

Arroyo et al. [41] showed that pollinator visitation rates in the Andes of central Chile are seldom consistent. For example, pollinator visitation rates to *Chaetanthera euphrasiones* capitula ranged from zero at low elevation (2810 m) to 0.12 visits per capitulum per 25 min at higher elevation (3315 m). Mean pollinator visitation rates to *C. renifolia* (LP = 0.079, PN = 0.047) were very similar to those reported by Torres-Díaz et al. [35] for cold microclimate populations of *C. apiculata* and *C. lycopodioides* (0.057 and 0.075 respectively), but lower than those of populations in a warmer habitat (0.446 and 0.307, respectively).

The percentage of achene set of *C. renifolia* was significantly constrained by pollen availability. This is consistent with the relatively low pollinator visitation rates found at both sites. In addition, we found evidence that supplemental pollen addition reduces the mass of individual achenes, which suggests that female reproductive output is constrained by resource availability. As achene mass is negatively correlated to achene germination [e.g. 42, 43], it is likely that there would be a cost of increasing achene output in years of abundant pollinators in this species. As shown by Torices & Méndez [44] achenes within a capitulum can compete by resources, therefore, if fewer achenes compete by resources these may achieve a larger size. In a recent meta-analysis, García-Camacho & Totland [33] showed that although alpine plants suffer significant pollen limitation, there is no difference in pollen limitation between alpine and lowland species, and between self-compatible and self-incompatible species. Although female reproductive success of *C. renifolia* was pollen-limited, the differences in pollinator visitation rates between populations were not translated into differences in achene output. Different insect species can drastically differ in their qualities as pollinators [45], [46]. Therefore, the marked differences in pollinator assemblage composition between LP and PN may be involved in the lack of differences in female reproductive success between sites despite contrasting visitation rates. For instance, although less visited, PN was mainly visited by *F. leucoglene* (Lepidoptera), which has the largest body size among the observed flower visitors (data not shown) and could have transferred higher pollen loads onto stigmas. However, further information is needed to validate this hypothesis.

Although a number of studies have shown that flowers of high-elevation plants usually receive fewer visits per time unit, they can compensate for the lack of pollination service by increasing its longevity with altitude [23]–[29], [47]. Floral life (and attraction) may conclude shortly after pollination [48] and varies widely among species [49]. For instance, several orchids wilt within a day or two after pollination but some species can maintain flowers for as much as nine months if unpollinated [50], [51]. Interestingly, pollinator-excluded *C. renifolia* capitula extended their stigma longevity for up to 25.8 and 26.8 days. Given that the male phase can extend for 7–9 days (data not shown), the total capitulum longevity in *C. renifolia* can be as long as ∼37 days, a remarkable feature considering the floral maintenance demands in such a low resource environment. The increased stigma longevity appears to be an adaption to the low availability of pollinators at high elevation. Ashman & Schoen [49] showed that there is a negative relationship between floral longevity and pollinator visitation rate. Our estimations of stigma longevity were obtained in the absence of pollinators, which provides a realistic estimation of the maximum potential capitulum longevity. Prolonged floral longevity seems to be common at high elevations. Arroyo et al. [37] found that capitula longevity increases from 4.1 days at 2310 m to 9 days at 3500 m in the Andes of central Chile. Primack [43] reported 6.9 days (ranging from 4 to 12 days) of flower longevity for 9 subalpine Chilean species. In turn, Fabbro & Körner [29] and Primack [43] reported slightly longer longevities for European alpine (8.3 days) and for subalpine species from New Zealand (7.8 days), respectively. Bingham & Orthner [24] found that while low-elevation populations of *Campanula* remained receptive for 1.5 days, high-elevation flowers were receptive for 2.4 days. It is important to note that our measures of stigma longevity are at inflorescence level (capitulum), whereas data from other authors are at flower level. To our knowledge, the stigma longevity reported in the present study has not been reported before in any alpine species. This prolonged stigma longevity may increase pollination opportunities for this high-elevation triennial species. Future studies should evaluate the limits of extended stigma lifespan in this alpine species.

### Concluding remarks

In a general context, our results add new evidence to a growing number of studies that emphasize that autogamous reproduction is far from being a rule in high elevation ecosystems. The extremely high stigma longevity found here appears to be an adaption to life at high elevation that can increase opportunities for cross-pollination.
